# The Effect of L-Hook Dissection and Scissors Dissection on Liver Injury in Laparoscopic Cholecystectomies

**DOI:** 10.7759/cureus.15245

**Published:** 2021-05-25

**Authors:** Murat Baki YILDIRIM, Murat B Ozkan, Ramazan Topçu

**Affiliations:** 1 General Surgery, Hitit University, Faculty of Medicine, Corum, TUR

**Keywords:** liver damage, safe laparoscopic cholecystectomy, gallbladder dissection, cautery damage, scissor

## Abstract

Purpose: Laparoscopic cholecystectomy is generally performed with the help of monopolar cautery. We aimed to reveal the effect of monopolar cautery use on liver damage in this study.

Method: Data of patients who underwent elective cholecystectomy between January 2016 and April 2020 were collected retrospectively. The patients were divided into two groups according to the surgical technique as hook dissection (HD) and scissor dissection (SD). The amount of increase in the preoperative and postoperative alanine aminotransferase (ALT) and aspartate aminotransferase (AST) values of the patients was compared between the two groups.

Findings: Over 970 patients were included in the study. The changes in pre-post ALT and AST values were statistically significantly different between the HD (n=469) and SD (n=501) groups (p<0.001; p0.001). ALT (26 (−25, 338)) and AST (27 (−23, 444)) changes in the HD method were statistically significantly higher than ALT (11 (−16, 371)) and AST (10.8 (−37, 617)) changes in the SD method.

Results: ALT and AST values increase after all laparoscopic cholecystectomies. Although the increase in ALT and AST in the HD patients is statistically significant when compared to the SD group, both methods of laparoscopic cholecystectomy can be safely performed because they do not cause permanent liver injury.

## Introduction

The first laparoscopic cholecystectomy (LC) was described by Dr. Mühe 35 years ago, and it has since evolved to become the gold standard for cholecystectomies [1]. Today, the rate of having elective cholesterol is 367 per 100,000 women, while it is 134 per 100,000 men [2]. Therefore, cholecystectomy is one of the most common procedures performed in the general surgery clinic. LC has advantages such as short hospital stay, low wound infection, improved cosmetic outcomes, and early return to work. However, LC has the potential for both preoperative and postoperative complications like any surgical procedure. Intraoperative complications can be listed as injuries to the great arteries and veins, bile duct injury, gallbladder perforations, and thermal damage to the liver, while postoperative complications are seen as wound infection, bile leakage, atelectasis, and deep vein thrombosis [3]. Impairment may occur in liver function tests (LFT) after LC. An increase of up to 80% can be seen in LFT in studies [4]. This increase may be due to the type and duration of the anesthetic agent administered, CO_2_ pneumoperitoneum, and bile duct injuries [5,6]. Besides, electrocauterization-induced damage to the liver bed can result in an increase in LFTs following surgery [7]. Alanine aminotransferase (ALT) and aspartate aminotransferase (AST) are two significant tests performed to evaluate LFT and directive in the detection of acute liver injury [8]. In today's LC surgeries, two methods are commonly used to separate the gallbladder from the liver. This study was conducted to investigate and compare the effects of two different dissection methods, hook dissection (HD) and scissor dissection (SD), on postoperative changes in LFT levels.

## Materials and methods

**Figure 1:**
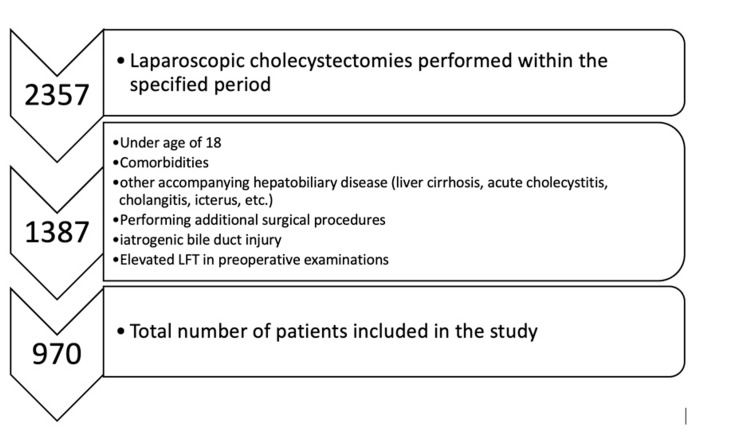
Flowchart: inclusion and exclusion criteria of the study

The data of patients who underwent LC at Hitit University Faculty of Medicine Training and Research Hospital between January 01, 2016, and April 30, 2020, were analyzed retrospectively. Before the study, ethics committee approval was obtained from Hitit University Clinical Research Ethics Committee with decision number 227. Information on age, gender, body mass index, surgery duration, surgical technique, and postoperative complications were obtained from the hospital database. It was found that 2357 patients underwent LC surgery during the study period. Patients under 18 years of age, or who had an additional disease (diabetes mellitus, hypertension, congestive heart failure, etc.), underwent cholecystectomy due to acute cholecystitis, had cholangitis or acute pancreatitis, an additional surgical procedure, obstructive jaundice, bile duct, duodenal or vascular injury during surgery, postoperative acute pancreatitis and needed additional postoperative medication, as well as pregnant women, were excluded from the study. ALT and AST values assessed in this study were measured at the postoperative twenty-fourth hour. In our institute, we routinely practice liver function enzymes and total blood count from patients postoperatively. For this reason, even if no complications were suspected, these values ​​could be reached in the database since LFTs were studied in all patients in the postoperative period. Furthermore, patients with ALT and AST elevation due to any reason in their preoperative examinations were excluded from the study. The study included 970 patients after the implementation of exclusion criteria (Figure 1). The patients were split into two groups based on how the gallbladder was dissected from the liver bed. The first group consisted of patients who had their gallbladder bed dissected with an L-hook (HD), while the second group consisted of patients who had their gallbladder bed dissected with laparoscopic scissors (SD). The technique of dissection was selected by the surgeon’s preference. In the SD group, surgeons did not use a hook dissector for the entire surgical procedure. In the HD group, during the separation of the gallbladder from the liver bed, monopolar cautery was used throughout the entire dissection, whereas in the SD group, it was observed that the dissection was performed by cutting with scissors and monopolar cautery was used only when necessary to achieve hemostasis. There is a significant difference between the two groups in terms of cautery usage because surgeons in the SD group use scissors to dissect and cut without electrocautery unless there is active bleeding. But hook dissector cannot be used without electrocautery because of its working principle. It was observed that the remaining steps of this surgical procedure were the same for all patients included in the study.

Statistical analysis in the study was performed using SPSS (Version 22.0, SPSS, Inc., Chicago, IL, USA, license: Hitit University) package program. Descriptive statistics for continuous data are reported with median (min-max) when data are not normally distributed, and normally distributed data are reported with mean ± standard deviation (SD). Descriptive statistics of categorical data were reported as number (n) and percentage (%). The normal distribution of data was analyzed using the Kolmogorov-Smirnov test. The Mann-Whitney U test was used to compare continuous variables between two independent groups since the data were not normally distributed. Wilcoxon signed-rank test was used for comparison before repeated measurements because the data were not normally distributed. The value for the statistical significance level was accepted as p<0.05.

## Results

A total of 970 patients were included in the study. The mean age of the patients was 51.62±13.78 (17-89) years. The gender, American Society of Anesthesiologist (ASA) scores, and body mass index (BMI) of patients in both groups were similar. The mean total duration of hospitalization of the patients was 3.77±2.43 (1-28) days. The mean duration of surgery was 41.62±20.99 (12-165) minutes. In 48.4% (n=469) of the patients, the HD method was used, while the SD method was used in 51.6% (n=501) of the patients. The comparison of patients' age, days of hospitalization, and duration of surgery between the groups are shown in Table 1. The mean age of the patients in the two groups were similar (p=0.188). The mean age of the group in which the HD method was used was 52.13±14.37 (median (min-max): 54 (17-86)) and the mean age of the group in which the SD method was used was 51.14±13.21 (median (min-max): 52 (19-89)). Hospitalization and surgery times were significantly higher in the second method (p<0.001, p<0.001, respectively, Table 1).

**Table 1:** Comparison of patients' ages, gender, body mass index, days of hospitalization, and duration of surgery between the groups

	HD method (n=469) mean ± SD	SD method (n=501) mean ± SD	P-value
Age	52.13±14.37	51.14±13.21	0.188
Gender: male/female	232 (%49,.5)	257 (%51.3)	0.569
	237 (%50.5)	244 (%48.7)	
BMI (kg/m^2^)	24.52±2.21 (24)	24.49±2.25 (25)	0.840
Duration of hospitalization	3.59±2.70	3.93±2.13	<0.001
Duration of surgery	38.90±18.77	44.17±22.60	<0.001

A comparison of pre-operative and post-operative values of ALT and AST measurements according to surgical methods is presented in Table 2. The preoperative and postoperative ALT and AST measurements were statistically significantly different as a result of the operation performed using both surgical methods (p<0.001; Table 2). The postoperative ALT and AST values were statistically significantly higher than the preoperative ALT and AST values in both surgical methods (Table 2).

**Table 2:** Comparison of pre-post values of ALT and AST measurements ALT: alanine aminotransferase, AST: aspartate aminotransferase, HD: hook dissection, SD: scissor dissection.

	HD method (n=469) mean±SD		P-value	SD method (n=501) mean±SD		P-value
	Pre-op	Post-op		Pre-op	Post-op	
ALT	35.38±6.18	61.54±17.05	<0.001	34.90±5.88	49.93±30.99	<0.001
AST	27.91±13.41	64.10±44.16	<0.001	26.96±12.93	51.32±66.15	<0.001

The comparison of ALT and AST difference values (post-pre changes) between the groups developed according to surgical methods is presented in Table 3. There were statistically significant differences in pre-post ALT and AST changes between surgical method groups (p<0.001; p<0.001). ALT (26 (−25, 338)) and AST (27 (−23, 444)) changes in the HD method group were statistically significantly higher than ALT (11 (−16, 371)) and AST (10.8 (−37, 617)) changes in the SD method group.

**Table 3:** Comparison of difference values (changes) of ALT and AST measurements between surgical method groups ALT: alanine aminotransferase, AST: aspartate aminotransferase, HD: hook dissection, SD: scissor dissection.

	HD method (n=469) mean±SD	SD method (n=501) mean±SD	P-value
Post-op ALT - Pre-op ALT	26.16±18.15	15.02±31.40	<0.001
Post-op AST - Pre-op AST	36.18±46.55	24.36±67.64	<0.001

## Discussion

One of the most efficient methods that show liver injury is the measurement of ALT and AST values from the blood, which are used for evaluating liver function. Measuring ALT and AST is both easy and non-invasive in comparison to other methods [9]. In studies conducted, an increase in ALT and AST levels of patients may occur after LC. Although the cause of this increase is not fully revealed, it is considered to be multifactorial. These markers may increase in response to the age of the patient, the prevalence of additional diseases, the type of anesthetic drugs used, the length of anesthesia, or a possible bile duct injury [4,10]. Surgery is insufficient to explain the discrepancy in increase observed since the age distribution in both groups in our study is equal (Table 1). Besides, since the same anesthetic agents were used in both groups, it was thought that they were ineffective in making a distinction between the groups.

ALT and AST levels were found to be significantly higher in the LC group in studies comparing laparoscopic cholecystectomy to open cholecystectomy. The abdomen is inflated with CO_2_ to a pressure of 14 mmHg during LC. This is much higher than the typical portal pressure of 8 mmHg. This occurrence was believed to have resulted in a reduction in blood flow in the portal vein, and accordingly, it caused ischemia in the liver [11,12]. Furthermore, ALT and AST levels increase as the laparoscopy time lengthens [13]. This is because ischemia time is prolonged. Both groups included in the study had increased ALT and AST levels, as expected since the LC method was used (Table 2). However, when the two groups were compared in terms of surgery time, ALT and AST increase in the first group was found to be significantly higher, despite the second group's surgery being significantly longer (SD group). Therefore, this increase cannot be explained by the duration of the surgery and the length of exposure to anesthetic agents.

The main difference between the two methods is how monopolar cautery is applied. Considering the operation method in patients in the first group (HD), monopolar cautery energy was used for a longer period of time. Both approaches are equally safe in terms of bile duct injuries and postoperative bleeding [14,15]. In the literature, studies comparing monopolar cautery and ultrasonic dissector revealed that the groups using cautery had a significantly higher increase in LFTs in the postoperative period [16]. Besides that, the ultrasonic dissector has benefits such as a shorter operation time, less bile duct injury, and a shorter stay in the hospital [17,18]. However, since the ultrasonic dissector is both costly and difficult to access, electrocautery is still used today. There is currently no research to shed light on the relationship between electrocautery usage time and liver damage in the literature. In this study, the first group (HD) had significantly higher postoperative ALT and AST levels than the second group (SD). The longer period of monopolar electrocautery used in the first group during surgery is thought to be the cause of this increase.

In various studies, it is shown that increased ALT and AST values return to normal 72 hours after laparoscopic operations and do not cause any hepatic failure [19,20]. ALT and AST values assessed in this study were measured at the postoperative twenty-fourth hour. Since this study was designed retrospectively, the difference between the groups would be observed more clearly with prospective studies including measurements that will cover the seventy-second hour.

Limitation

ALT and AST levels could be more elevated in cases in which the gallbladder was densely attached to the liver. There is no record of the difficulty levels of cholecystectomies in the data system, and because this study carried out retrospectively, these data cannot be reached.

## Conclusions

ALT and AST values increase after all laparoscopic cholecystectomies. HD causes more electrothermal damage. Although the increase in ALT and AST in the HD patients is statistically significant when compared to the SD group, both methods do not cause permanent liver injury. Also, the presented data are insufficient to conclude that HD leads higher elevation of AST and ALT than SD, as several confounding factors were not taken into consideration. Further randomized studies which standardize the confounding factors should be done to prove the difference between dissection techniques prospectively.
